# Characterization of *Aeromonas* Isolates from Ornamental Fish: Species, Virulence Genes, and Antimicrobial Susceptibility

**DOI:** 10.3390/microorganisms12010176

**Published:** 2024-01-16

**Authors:** Carolina H. de Oliveira, Luisa Z. Moreno, Pedro H. M. Cardoso, Ana Paula S. Silva, Vasco T. M. Gomes, Mikaela R. F. Barbosa, Simone C. Balian, Andrea M. Moreno

**Affiliations:** 1 Department of Preventive Veterinary Medicine and Animal Health, School of Veterinary Medicine and Animal Science, University of São Paulo, Av. Prof. Dr. Orlando Marques de Paiva 87, São Paulo 05508-270, SP, Brazil; ch-oliveira@outlook.com.br (C.H.d.O.); luzanolli@gmail.com (L.Z.M.); pedrohenriquemedvet@usp.br (P.H.M.C.); anapaula_silva2006@yahoo.com.br (A.P.S.S.); vaskotulio@yahoo.com.br (V.T.M.G.); balian@usp.br (S.C.B.); 2 Division of Microbiology and Parasitology, Department of Environmental Analysis, Environmental Company of the State of São Paulo (CETESB), Av. Prof. Frederico Hermann Júnior 345, São Paulo 05459-900, SP, Brazil; mrbarbosa@sp.gov.br

**Keywords:** *Aeromonas* spp., ornamental fish, PCR, antimicrobial resistance, AFLP

## Abstract

This study aimed to characterize 300 *Aeromonas* spp. strains isolated from 123 ornamental fish of 32 different species presenting with septicemia, skin lesions, and/or eye lesions. Within the 300 strains, 53.0% were identified as *A. veronii*, 41.3% as *A. hydrophila*, and 5.7% as *A. caviae*. Among the six virulence genes investigated, the most frequent were *act* (90.3%) and *aer* (79.3%). More than 50% of *A. hydrophila* strains were positive for all the studied genes. A total of 30 virulence profiles were identified, with the five main profiles identified comprising 75% of strains. Only five strains were negative for all genes and were identified as *A. caviae* and *A. veronii*. The antimicrobial susceptibility profile was performed for 234 strains, with sulfonamides presenting more than 50% of the resistance rates. Susceptibility was observed mainly for cephalosporins, aminoglycosides, chloramphenicol and piperacillin-tazobactam. Multidrug resistance was detected in 82.5% of the studied strains, including *A. caviae* with 100% multidrug resistance, and *A. hydrophila* with 90.9% multidrug resistance. The SE-AFLP analysis resulted in 66 genotypes of *A. hydrophila*, 118 genotypes of *A. veronii*, and 14 genotypes of *A. caviae*, demonstrating the greater heterogeneity of *A. veronii* and A. *caviae*. However, no direct correlation was observed between the genotypes and the strains’ origins or virulence and resistance profiles.

## 1. Introduction

The aquaculture industry has developed rapidly in recent decades and the ornamental fish industry has contributed to this growth. Ornamental fish are valued for their beauty and distinctive colors; however, the high demand for these animals means that their commercialization is constantly growing. In parallel with the intensification of ornamental fish production, there is an increase in infectious diseases [1].

Over the last two decades, the expansion of aquaculture has provided a satisfactory understanding of the pathogenesis of bacterial diseases in fish. However, it is still considered insignificant when compared to knowledge about bacterial diseases in other animals and humans [2].

High levels of morbidity and mortality caused by bacterial infections can be found in farmed fish [2], and is acknowledged as a serious problem for the ornamental fish industry [1]. Furthermore, these bacteria are also present in the microbiota of fish and water, being characterized as opportunistic pathogens [3].

Among these pathogens, the *Aeromonas* genus stands out. There are currently 36 species described in the genus, among which *A. hydrophila*, *A. veronii* and *A. caviae* are the most described in fish diseases [4,5]. Even though the advancement of molecular and phylogeny methods were essential for the establishment of a recent taxonomy, there is still controversy regarding the best technique for the rapid and assertive identification of these species [4,5].

Bacteria of the *Aeromonas* genus are commonly found in aquatic environments, and can also be isolated from various animal species, foods, and soil [6]. Aeromonosis in fish is transmitted horizontally from excreta or skin lesions, and can cause hemorrhagic septicemia and ulcerative disease syndrome, affecting any fish species [3]. High mortality can be observed between two and ten days after the onset of clinical signs, leading to extensive economic losses in the industry [7,8].

The *Aeromonas* species can be considered primary or opportunistic agents affecting immunocompromised fish and humans [6,9]. As part of the microbiota of fish and aquatic environments, these bacteria are associated with the disease mainly in fish raised in farms or in water reuse systems and in situations of stress and inadequate management [10].

Considering the financial and health importance of aeromonosis in aquaculture, the rapid and reliable detection of *Aeromonas* species, as well as their pathogenic potential, is of great interest to the sector. Similarly, the characterization of antimicrobial resistance profiles is also highlighted, considering the problem of the low number of antibiotics authorized for use in fish.

Therefore, the aim of the present study was to identify and determine the resistance and genotypic profiles of *Aeromonas* isolates from ornamental fish, through matrix-assisted laser desorption/ionization-time of flight (MALDI-TOF) mass spectrometry, PCR for species confirmation and virulence genes detection, the disc diffusion susceptibility test, and single enzyme amplified fragments length polymorphism (SE-AFLP).

## 2. Materials and Methods

### 2.1. Bacterial Strains

A total of 300 *Aeromonas* spp. strains were studied. These were previously isolated from 123 fish of 32 distinct species between the years of 2016 to 2019; these animals presented septicemia, skin lesion and/or eye lesions (Figure S1). This study was approved by the Animal Use Ethics Committee (CEUA) of the School of Veterinary Medicine and Animal Science, University of São Paulo, under the CEUA Process Number 6065110518.

The strains were isolated from samples of the affected organ/tissue of the fish or from superficial swabs (ocular and skin lesion swabs). The swabs were placed directly in Amies culture medium (ABSORVE™, CRAL, Cotia, Brazil), while the fish were transported in appropriate bags to the laboratory and samples were then collected under aseptic conditions and placed in sterile plastic bags for further processing. The fish and swabs were kept refrigerated (4 °C) until their arrival at the laboratory for processing (up to 72 h). For bacterial isolation, organ fragments or swabs were plated on MacConkey agar and dextrin-ampicillin agar (Difco, Sparks, MD, USA). The plates were incubated in aerobiosis, at 37 °C for 24 to 48 h. The characteristic isolated bacterial colonies (mostly non-lactose fermenter in MacConkey agar, and yellowish colonies in dextrin-ampicillin agar) were inoculated in 3 mL of BHI (Brain Heart Infusion) broth (Difco, Sparks, MD, USA) and from this culture an aliquot (700 µL) was separated for stock at −86 °C with sterile glycerol (300 µL).

### 2.2. Strains Reactivation and Hemolysis Evaluation

For strain reactivation, stock aliquots of the isolated strains (described above) were thawed. To check purity and evaluate hemolytic activity, the strains were re-isolated on MacConkey agar and blood agar (5% sheep blood) (Difco, Sparks, MD, USA). The plates were incubated at 37 °C for 24 to 48 h in aerobiosis. For hemolysis evaluation, the blood agar plates were observed through transmitted light. Hemolysis was classified as: Beta hemolysis (β) when complete lysis was observed as clear transparent zone surrounding the colonies; Alpha hemolysis (α) when incomplete lysis was detected as a greenish discoloration of the medium surrounding the colonies; and Gamma hemolysis (γ) in the absence of lysis characterized by no alteration of the medium.

The isolated bacterial colonies were sub-cultured in 2 mL of BHI broth (Difco, Sparks, MD, USA) and further aliquoted for MALDI-TOF MS, DNA extraction and the Kirby–Bauer disk diffusion susceptibility test.

### 2.3. MALDI-TOF MS Identification

For MALDI-TOF MS identification, ribosomal protein extraction was performed as described by Hijazin et al. [11]. The protein spectra were captured by a Microflex™ mass spectrophotometer (Bruker Daltonics, Inc., Billerica, MA, USA) and FlexControl™ v3.4 (Bruker Daltonics, Inc., Billerica, MA, USA) software using the MTB_autoX method. The spectrophotometer was externally calibrated using the Bacterial Test Standard (BTS—Bruker Daltonics, Inc., Billerica, MA, USA). The microbial identification was performed by BioTyper™ 3.0 (Bruker Daltonics, Inc., Billerica, MA, USA) using the manufacturer’s criteria: the species were assigned with log score values ≥ 2.0; scores ≥ 1.7 and <2.0 determined only genus identification.

### 2.4. Molecular Identification and Virulence Genes Detection

The bacterial DNA was extracted according to the Boom et al. [12] protocol and maintained at −20 °C until processing. For species confirmation, the following genes were evaluated: *gyrB* for *A. caviae* detection [13], *ahaI* for *A. hydrophila* [14], and *rpoB* for *A. veronii* [13] (Table S1).

PCR was also applied for the screening of six virulence genes, including genes that encode heat-labile cytotonic enterotoxin (*alt*), aerolysin (*aer*), cytotoxic enterotoxin (*act*), heat-stable cytotonic enterotoxin (*ast*), flagellin (*fla*) and hemolysin (*hlyA*). The respective primers are presented in Table S1, and the reaction parameters were adjusted as described by Khor et al. [15].

The C1000™ Touch Thermal Cycler (Bio-Rad Laboratories, Hercules, CA, USA) was used and the PCR reactions (50 µL) comprised 5 µL of genomic DNA, ultrapure water, 10X PCR buffer, 1.5 mM MgCl2, 200 µM of dNTPs, 20 pmol of each primer, and 1 U of HOT FIREPol DNA-polymerase (Solis BioDyne, Tartu, Estonia). Amplicons were detected by agarose gel electrophoresis (1.5%) stained with BlueGreen™ (LGC Biotecnologia, Cotia, Brazil). Images were captured under UV illumination by the Gel Doc XR system (Bio-Rad Laboratories, Hercules, CA, USA) and the 100 bp DNA Ladder molecular weight marker (New England BioLabs Inc., Ipswich, MA, USA) was used for further band analysis.

### 2.5. Single Enzyme Amplified Fragments Length Polymorphism (SE-AFLP)

The SE-AFLP was carried out according to the McLauchlin et al. [16] protocol, with unique restriction by *HindIII* (New England Biolabs). The DNA fragments were detected with electrophoresis at 90 V for 4 h in 2% agarose gel stained with BlueGreen™ (LGC Biotecnologia, Cotia, SP, Brazil) and images were captured under UV illumination by the Gel Doc XR system (Bio-Rad Laboratories, Hercules, CA, USA). The amplified fragments were identified based on the molecular weight marker 100 pb DNA Ladder (New England BioLabs Inc., Ipswich, MA, USA).

### 2.6. Antimicrobial Resistance Profiling

The antimicrobial resistance profiling was carried out using the Kirby–Bauer disk diffusion technique, according to the standards defined in document VET01-S2 [17].

For inoculum preparation, the strains were cultivated in BHI broth (Difco, Sparks, MD, USA) and incubated at 37 °C for 24 h. The turbidity of the culture was adjusted with sterile saline solution (0.9%) to obtain an optical turbidity equivalent to 0.5 on the McFarland standard (approximately 1 × 10^8^ CFU/mL). Once adjusted, the bacterial suspension was homogeneously distributed, with the aid of a sterile swab, on a Mueller Hinton agar plate (Difco, Sparks, MD, USA). Subsequently, the antibiotics discs were aseptically applied, and the plates were incubated at 35 °C for 24 to 28 h, as previously described [18]. The *Escherichia coli* ATCC™ 25922 (ATCC, Gaithersburg, MD, USA) was used as an internal quality control.

The antimicrobials evaluated and their respective cut-off points are presented in Table S2. The resistance profile of the studied strains was determined based on the cut-off points available in CLSI documents M100, VET01S and VET04 [17,19,20]. Multidrug resistance classification was determined as described by Schwarz et al. [21], considering resistance to three or more antimicrobial classes. For enrofloxacin, florfenicol and gentamicin, the results were also evaluated using the epidemiological cut-off points (ECOFF) according to the VET04 document [19] (Table S2).

### 2.7. Statistical Analysis

The distribution of strain frequencies according to species and origin was carried out using the SPSS 16.0 program (IBM SPSS Inc., Chicago, IL, USA). To evaluate the agreement between MALDI-TOF MS and PCR techniques for *Aeromonas* species identification, the Kappa coefficient (k) and the symmetry and homogeneity test were performed with Stata 12.0 (StataCorp LLC, College Station, TX, USA). The identified species and the hemolysis phenotype were used as categorical variables, and the differences were analyzed using the Fisher–Freeman–Halton test with two-sided probability estimated using the Monte Carlo method. For the analyses carried out, a significance level of 5% was considered.

The Bionumerics 7.6 (Applied Maths, bioMérieux, Sint-Martens-Latem, Belgium) software was used for the SE-AFLP cluster analysis. A dendrogram was constructed for each *Aeromonas* species using the Dice coefficient and the UPGMA method (unweighted pair group method with arithmetic mean). To distinguish genotypes, the cut-off point of 90% of genetic similarity was applied [22].

## 3. Results

The studied *Aeromonas* strains were isolated from 123 ornamental fish of 32 distinct species, with a predominance of *Carassius auratus* (Linnaeus, 1758) (goldfish) (23.6%) and *Cyprinus carpio* var. koi (koi carp) (13.0%). From the 300 studied strains, 235 (78.0%) were isolated in 2016, 13 (4.3%) in 2018, and the remaining 52 strains (17.0%) were obtained in 2019. An average of 2.5 strains per animal was evaluated, considering the possibility of variations in isolation sites and *Aeromonas* species.

The MALDI-TOF MS identification resulted in 158 (52.67%) strains of *A. veronii*, 125 (41.67%) of *A. hydrophila*, and only 17 (5.67%) of *A. caviae* (Table 1). The molecular identification, on the other hand, resulted in 53.0% (159/300) of *A. veronii* strains, 41.3% (124/300) of *A. hydrophila*, and 5.7% (17/300) of *A. caviae* (Table 1). Even though occasional discrepancies between the techniques for the identification of these *Aeromonas* species were observed, no significant difference was detected between them (asymptotic symmetry test probability *p* = 0.7461); and they also showed good agreement according to the Kappa coefficient (Kappa = 0.731; *p* < 0.001).

Interestingly, the results showed that, in 67.5% (83/123) of the fish, a single *Aeromonas* species was recovered by culture, while 30.9% (38/123) presented two species, and in only two animals (1.6%) were three species were detected simultaneously. Based on these results, animal infection profiles were proposed considering the three identified species of the *Aeromonas* genus. Thus, seven infection profiles (P1 to P7) were identified (Table 2), of which P1, P2 and P4 are highlighted to present the highest frequencies corresponding, respectively, to the species *A. veronii* and *A. hydrophila* detected separated and concomitantly.

From the identification of the *Aeromonas* strains, species distribution in relation to origin was also evaluated, considering the affected fish species (Table 3) and their clinical conditions (Table 4). Among these results, it is noteworthy that 70.6% of the *A. caviae* strains were detected in animals presenting septicemia with or without associated ocular lesions. Similarly, 64.7% of *A. caviae* strains were isolated from organ pool and eye samples.

Regarding the evaluation of hemolytic activity, it was observed that 86.0% of the studied strains presented beta-hemolysis, while 14.0% showed alpha-hemolysis. The distribution of *Aeromonas* species in relation to hemolytic activity resulted in a statistically significant difference (*p* < 0.001), which was due to the predominance of *A. veronii* and *A. hydrophila* strains that presented a beta-hemolysis phenotype.

**Figure 1:**
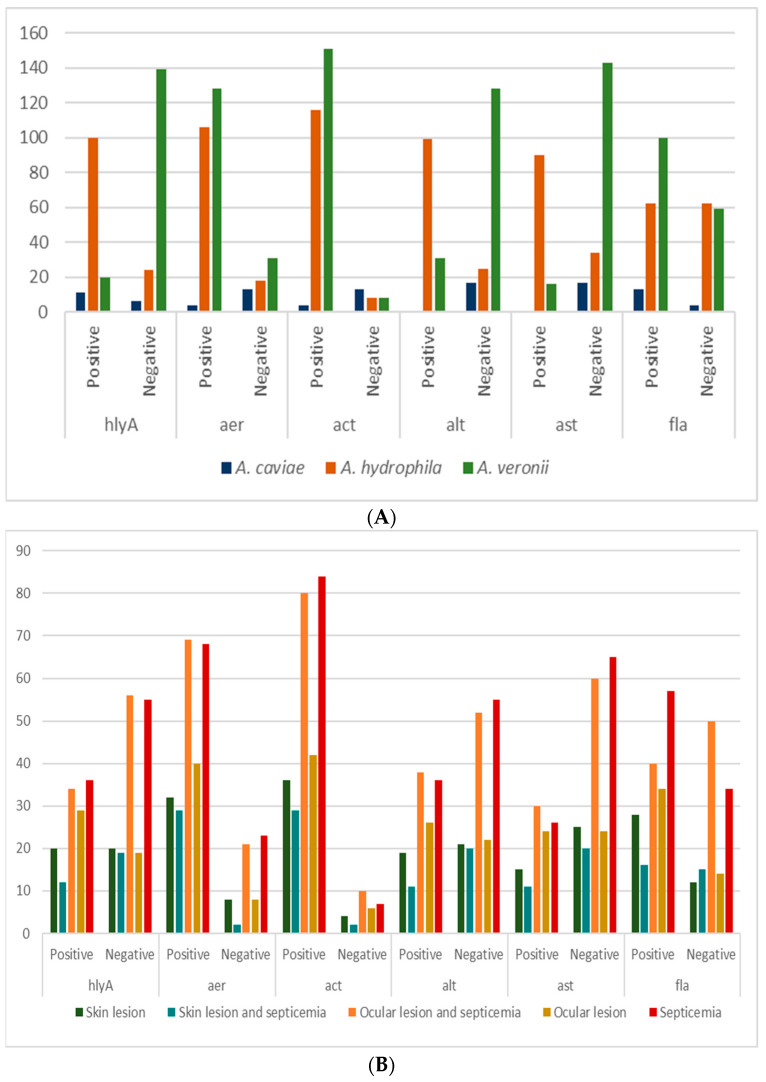
Representative graph of the distribution of virulence genes detected according to: (**A**) *Aeromonas* species; and (**B**) fish clinical conditions. Absolute number of isolates (*Y* axis) per virulence genes detected (*X* axis).

Of the six virulence genes investigated, the following results were observed: the *act* gene was detected in 271 (90.3%) strains, *aer* was detected in 238 (79.3%), *fla* in 175 (58.3%), *hlyA* in 131 (43.7%), *alt* in 130 (43.3%), and the *ast* gene was detected in 106 (35.3%). The species *A. hydrophila* strains were more than 50% positive for the studied genes, while *A. veronii* strains presented a higher proportion of *aer*, *act* and *fla*. Among the 17 *A. caviae* strains, there was a predominance of *hlyA* and *fla* genes (Figure 1A).

A significant statistical difference was detected for the beta-hemolysis phenotype and the genes *hlyA* (*p* < 0.001), *act* (*p* < 0.001), *alt* (*p* < 0.001), *ast* (*p* < 0.001), and *fla* (*p* = 0.029). Despite a tendency towards predominance of the *aer*, *act* and *fla* genes in relation to the clinical conditions of the fish (Figure 1B), a significant statistical difference was only detected for the *fla* gene (*p* = 0.08).

A total of 30 virulence profiles were identified considering the combination of PCR results (Table 5). It is noteworthy that the five main profiles contained 227 strains (75.7%) (profiles V1 to V5). Only five strains (1.7%) were negative for all genes (V10 profile), with these being identified as *A. caviae* and *A. veronii*; while 31 strains (10.3%) were positive for all genes (V4 profile) of which 30 were *A. hydrophila*. No relationship was observed between virulence profiles and the clinical conditions of the fish.

**Figure 2:**
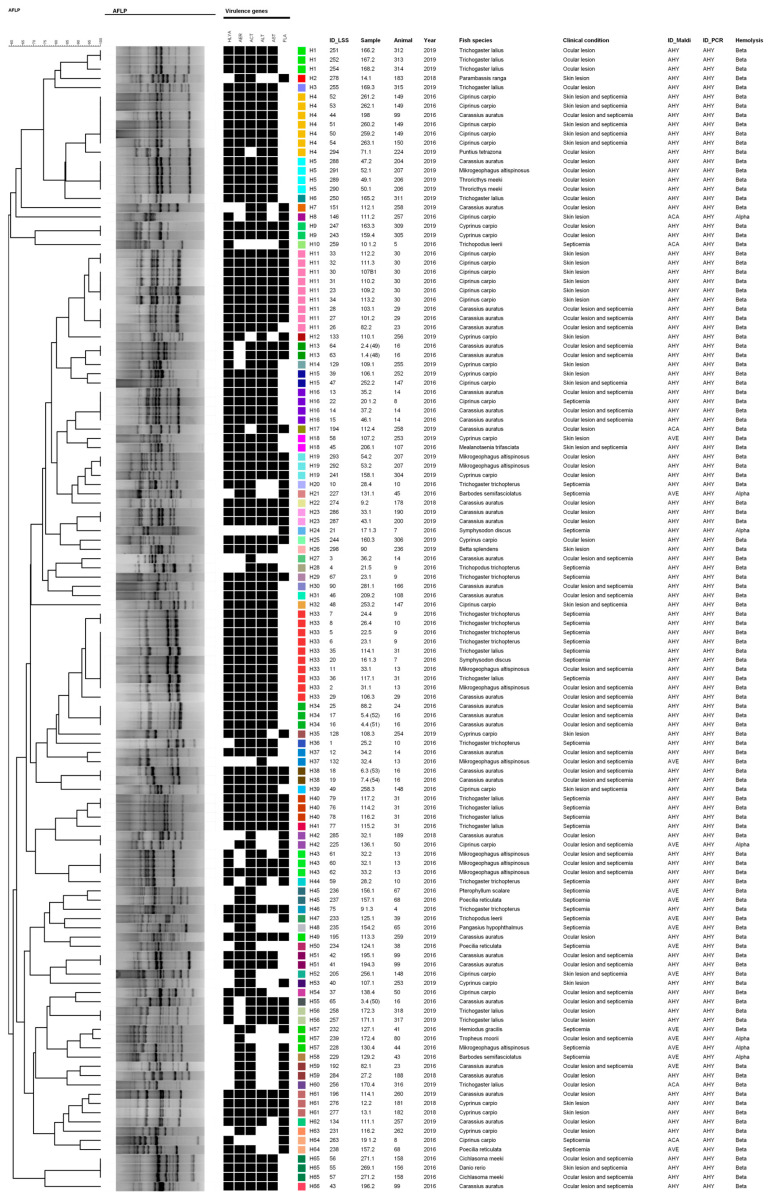
Dendrogram of SE-AFLP genetic profiles identified for *A. hydrophila* strains. Black squares represent presence of virulence gene; colored squares represent the identified genotypes.

**Figure 3:**
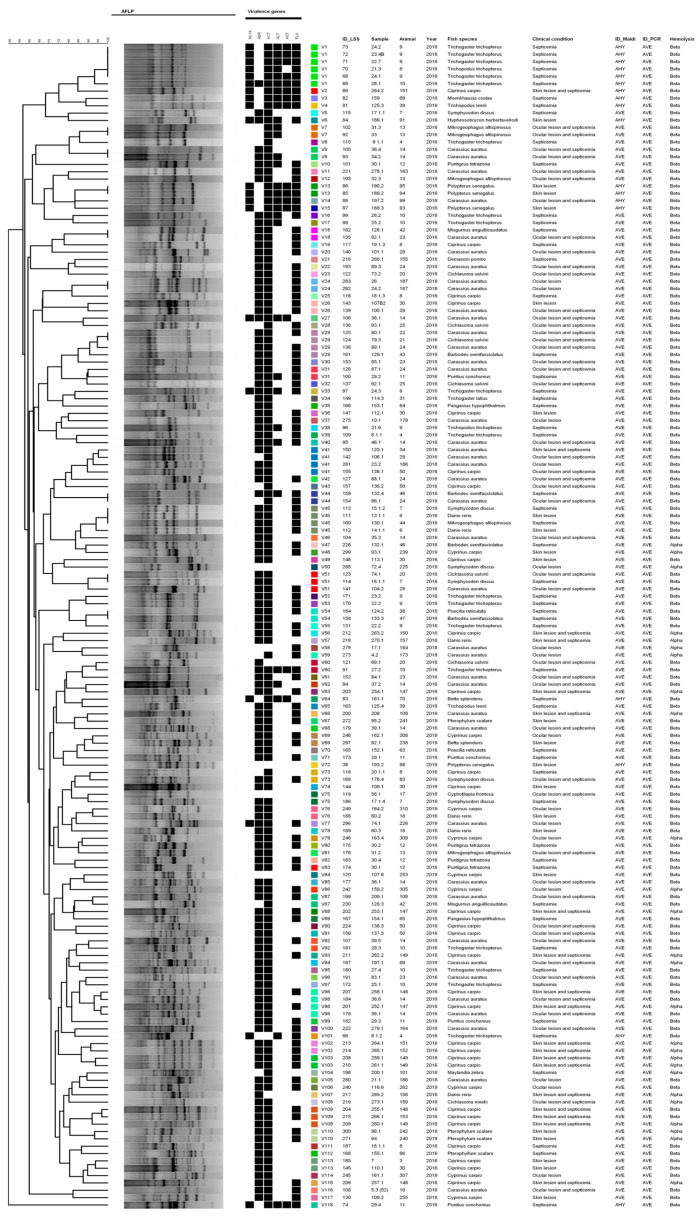
Dendrogram of SE-AFLP genetic profiles identified for *A. veronii* strains. Black squares represent the presence of a virulence gene; colored squares represent the identified genotypes.

**Figure 4:**
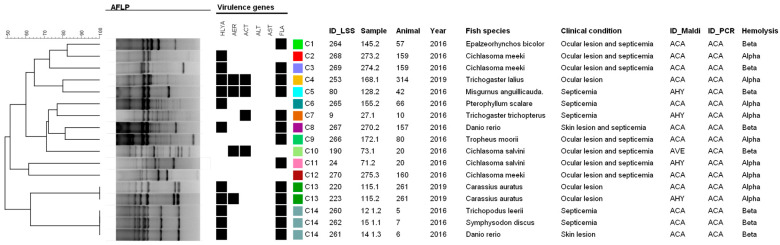
Dendrogram of SE-AFLP genetic profiles identified for *A. caviae* strains. Black squares represent the presence of virulence gene; colored squares represent the identified genotypes.

The SE-AFLP analysis was performed separately for each *Aeromonas* species (Figure 2, Figure 3 and Figure 4). For *A. hydrophila* (Figure 2), 66 genotypes (H1–H66) were identified among the 124 studied strains. No direct correlation was observed between the genotypes and the origin of the strains or virulence profile. However, the following results stand out: (1) clonal profiles related to different colonies or samples from the same animal (H13, H38, H43, H40, H43, H51); (2) clonal profiles composed of different animals of the same species from an aeromonosis outbreak with the same or different clinical signs (H1, H9, H23, H34, H56); and (3) clonal profiles composed of animals from different species from an aeromonosis outbreak (sharing an aquarium or water source) with the same or different clinical signs (H4, H5, H11, H33, H65).

The *A. veronii* dendrogram resulted in 118 genetic profiles (V1–V118) among the 159 strains studied (Figure 3), demonstrating great heterogeneity of the species. Similar to *A. hydrophila* genotypes, *A. veronii* genetic profiles composed of different animals of the same species were observed, as well as clonal profiles composed of animals of different species; however, *A. veronii* strains presented a greater difference or distance in the time of isolation when compared to *A. hydrophila*.

In relation to *A. caviae*, 14 SE-AFLP genotypes (C1–C14) were identified for the 17 strains studied (Figure 4), also showing greater variability of the species when compared to *A. hydrophila*. In this case, only two clonal profiles were identified: C13, composed of two colonies of goldfish A261, and C14, composed of three animals from different species from an outbreak of aeromonosis. Similar to the other studied species, no association was observed between the genotypes and the identified virulence profiles.

Antimicrobial resistance profiling was carried out for 234 strains (78.0%), as 66 strains were not recovered in the reactivation process to perform the disk diffusion technique. Of the recovered strains, 15 (6.4%) were *A. caviae*, 77 (32.9%) were *A. hydrophila*, and 142 (60.7%) were *A. veronii*.

Among the 16 antimicrobials evaluated, sulfonamide and sulfamethoxazole-tripmethopim showed, respectively, 92.7% and 50.4% resistance rates (Table 6). Erythromycin, imipenem, and ciprofloxacin presented 74.8%, 41.9% and 38.0% of the strains with intermediate results. Susceptibility was observed mainly for cephalosporins (all those evaluated showed more than 75% sensitivity), aminoglycosides (with gentamicin and amikacin having 86.8% and 79.5% sensitivity, respectively), and chloramphenicol and piperacillin-tazobactam having sensitivity greater than 88%. For enrofloxacin, florfenicol and gentamicin, the results from the epidemiological cut-off points (ECOFF) are presented in Table 7; there is a predominance of non-wild-type strains (NWT) for the three antimicrobials.

**Figure 5:**
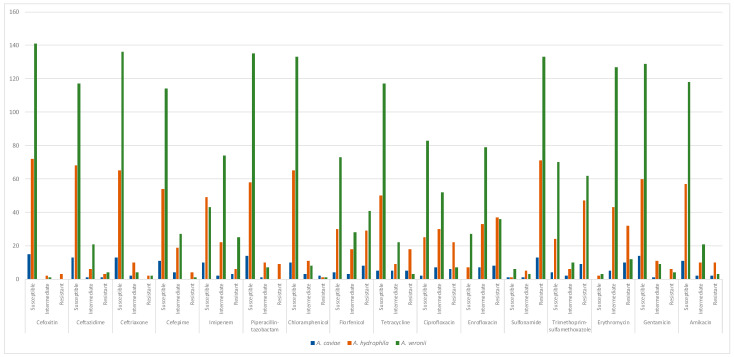
Representative graph of antibiogram results observed according to *Aeromonas* species. Absolute number of isolates (*Y* axis) per susceptibility results (*X* axis) for each *Aeromonas* species.

Multidrug resistance was detected in 82.5% of the strains studied, with *A. caviae* strains surprisingly presenting 100% multidrug resistance. *A. hydrophila* presented 90.9% multidrug resistance, followed by *A. veronii* strains, with 76.0%. Figure 5 presents the antibiogram results according to the *Aeromonas* species. No differences were observed between the resistance profiles for the studied *Aeromonas* species.

## 4. Discussion

For over 20 years, the taxonomy of the *Aeromonas* genus has been updated as a result of the application of molecular techniques and phylogenetic analyses; these genotypic analyses significantly differ compared with traditional phenotypic identification [7]. Therefore, the correct identification of these bacteria at the species level is still a challenge for most clinical microbiology laboratories [24].

Recently. MALDI-TOF mass spectrometry was considered an alternative technique for identifying *Aeromonas* strains [25]. The MALDI-TOF MS presents high precision and rapid results; however, as it is a technique dependent on prior bacterial isolation, may present variations in the species identified due to the culture media and growth conditions used [26]. Furthermore, considering that the technique is mainly based on the detection and differentiation of ribosomal protein peaks, it is known that MALDI-TOF MS has limitations when distinguishing very closely related species due to the high similarity of ribosomal compositions [27].

This situation has already been reported for the *Aeromonas* and *Edwardisella* genera, which present low specificity in species identification by MALDI-TOF mass spectrometry due to the low heterogeneity of ribosomal composition [26,27,28]. Despite this, the technique can still be used in routine diagnoses with reliable results for genera identification.

The results obtained in the present study corroborate the information described above. Although MALDI-TOF MS presented good agreement with the molecular identification carried out, it was observed that in more than 90% of the studied strains, the technique resulted in more than one identification match for two different *Aeromonas* species, with scores ≥ 2.0. Thus, despite speeding up the diagnosis, the mass spectrometry results also raise doubts regarding the adequate assignment of species within the *Aeromonas* genus.

*Aeromonas* species will often cause damage to hosts after injury or stress [29]. Janda and Abbott [6], after decades of studying the *Aeromonas* genus, reported that the species commonly isolated from clinical cases involving extra-intestinal and systemic infections in humans are *A. hydrophila*, *A. veronii* and *A. caviae*. In our study, we found the same species causing clinical disease in ornamental fish. For a long time, *A. hydrophila* was the main agent found in sick fish; however, recent reports involving *A. veronii* have increased [29]. Corroborating this recent increase in cases related to *A. veronii*, 53.0% (159/300) of the strains isolated in the present study were identified as *A. veronii*, followed by 41.3% (124/300) as *A. hydrophila*, with the remaining strains 5.7% (17/300) identified as *A. caviae*.

Regarding the hemolytic characteristics of *Aeromonas* strains, they can present two types of hemolysins without enterotoxic properties: α-hemolysins and β-hemolysins, responsible for the osmotic lysis of erythrocytes [8]. Although there is no clear relationship between *Aeromonas* species and the hemolytic phenotype, Nakano et al. [30] found that, among the strains isolated from aquatic environments (marine and riverine surface waters), the majority of *A. hydrophila* strains were highly hemolytic, while only 11% of *A. caviae* strains presented a hemolytic phenotype. More recently. in a Brazilian study with 117 *Aeromonas* strains isolated from vegetables, water, and feces from patients with diarrhea, Castilho et al. [31] detected 100% of strains with a beta-hemolysis phenotype. In the present study, however, a predominance of beta-hemolytic strains was observed for *A. veronii* and *A. hydrophila*, while 52.9% of *A. caviae* strains (9/17) presented an alpha-hemolysis phenotype.

Năcescu et al. [32] were the first to propose an association between the hemolytic activity of *Aeromonas* strains and their pathogenic potential. According to Heuzenroeder et al. [33], the presence of the *hlyA* and *aer* genes in *A. hydrophila* strains makes them more virulent and capable of causing diarrhea regardless of their origin of isolation. It is also known that other cytotoxins, such as cytotoxic enterotoxin (encoded by the *act* gene), may participate in the pathogenesis of *Aeromonas* infection [34]. Our results corroborate these data, with hemolytic strains presenting higher positivity for *act* (241/258) and *aer* (209/258) genes.

Guerra et al. [35], working with *Aeromonas* strains isolated from patients with gastroenteritis in southern Brazil, found that *A. hydrophila* and *A. veronii* species presented more virulence genes when compared to *A. caviae* strains. A similar result was observed in the present study, in which more than 50% of *A. hydrophila* strains were positive for the evaluated genes. *A. veronii* strains showed a higher proportion of *aer*, *act* and *fla*, while *A. caviae* strains showed positive results for the *hlyA* and *fla* genes. Also noteworthy was the presence of five strains of *A. veronii* and *A. caviae* that were negative for all studied genes, in contrast. 31 *A. hydrophila* strains were positive for all these genes. These data reveal that there is a difference between *Aeromonas* species in relation to the presence of virulence genes and that the species may present different mechanisms to infect the host, as proposed by Khor et al. [15].

Regarding the SE-AFLP analysis, our findings demonstrate that *Aeromonas* strains have great genetic variability, especially *A. caviae* strains, in which 14 genotypes were identified for the 17 strains studied. For *A. veronii* strains, we also noted important genetic heterogeneity, with 118 genetic profiles identified among the 159 strains studied. In the present study, no direct relation was observed between the genotypes and the origin or virulence profile of the *Aeromonas* strains. This differs from results obtained by Lund et al. [36], in which genetic clusters indicated a relationship between the *A. salmonicida* isolates and the fish host species. Pablos et al. [37] were also able to correlate the transmission of *A. caviae* through contaminated water and disease in humans through genotyping. However, both studies applied the amplified fragment length polymorphism using two restriction endonucleases that may have enhanced the technique’s discriminatory power.

The exchange of genetic information between bacteria of the *Aeromonas* genus has already been reported as a public health problem [38] because it allows for the emergence of multi-resistant strains. Furthermore, the aquatic environment receives daily urban and industrial effluents containing medicinal residues that will favor the emergence of strains more resistant to commercial antimicrobials.

According to Zhu et al. [39], enrofloxacin is one of the main antimicrobials used in aquaculture, and, despite its high potency, *Aeromonas* strains were recently shown to have resistance to this fluoroquinolone. Our results are in agreement with this report, with a notable 34.6% resistance and 50.9% intermediate susceptibility to enrofloxacin. These data are important because they demonstrate that even though its use is not licensed for aquatic animals in Brazil, the indiscriminate use of enrofloxacin is promoting an increase in resistance in *Aeromonas* strains. Furthermore, the data suggest the need for more studies involving antimicrobials and aquatic environments.

Similarly, an increase in resistance to florfenicol was also reported by Zhao et al. [40] due to its rising and improper usage in aquatic environments. In our work, we found a resistance rate of 33.4% and intermediate susceptibility of 20.9% for this antimicrobial. This demands attention, considering that florfenicol is one of the few antimicrobials licensed for use with ornamental fish.

Tetracyclines, on the other hand. are used worldwide in the treatment of animals from aquatic environments. and their use is permitted in countries such as the United States of America. the Czech Republic. Republic of Korea and Japan [41]. In Brazil, its use, together with florfenicol, is licensed for fish farming [42]. Therefore, the detection of 73.5% susceptibility for tetracycline in the present study demonstrates that this continues to be a good antimicrobial choice for ornamental fish. Sharma et al. [43] also reported tetracycline susceptibility in *Aeromonas* strains from *Clarias magur* (Hamilton, 1822), a fish species native to Southeast Asia. However. Hossain et al. [44] detected more than 70% resistance to tetracycline in *Danio rerio* (Hamilton, 1822) (zebrafish) in Republic of Korea. Therefore, as it is an important antimicrobial for fish farming, the use of tetracyclines must be monitored, including for ornamental systems.

Erythromycin and imipenem are antimicrobials used to treat humans. Dhanapala et al. [45], in a study with ornamental fish in Sri Lanka, found erythromycin resistance in 26.1% of 161 *Aeromonas* strains and 18% resistance to imipenem. These results corroborate those of the present study, in which resistance rates of 23.1% to erythromycin and 14.5% to imipenem were observed. However, we also observed 74.8% and 41.9% intermediate susceptibility to these antimicrobials, indicating an increase in their resistance in the near future.

Batra et al. [46] already described *Aeromonas* susceptibility to aminoglycosides, tetracyclines, amphenicols, quinolones, cephalosporins, carbapenems, monobactams and piperacillin. Here, we also found an over 76.5% susceptibility for all the aminoglycosides and cephalosporins tested, corroborating the reported data. The piperacillin–tazobactam combination showed an 88.5% sensitivity, which is also in agreement with the abovementioned study. Specifically, for florfenicol, gentamicin and enrofloxacin, the ECOFF cut-offs enables the verification, between wild type and non-wild type populations, of the presence or absence of acquired resistance and mutational mechanisms [47]. Our results demonstrate that more than 50% of the studied *Aeromonas* strains are classified as non-wild type; therefore, their resistance to these antimicrobials is related to acquired mechanisms.

Multiresistance was detected in 82.5% of the studied strains, with the *A. caviae* strains standing out with 100% multidrug resistance, followed by *A. hydrophila* with 90.9%, and *A. veronii* with 76.0%. Hossain and Heo [41] also reported similar results, in which multi-resistance was detected in more than 70% of the studied strains. Our results underline the warning made by the authors about increasing antimicrobial resistance and its association with improper and indiscriminate use in aquatic animals, especially in ornamental fish.

Finally, the results found in the present study demonstrate the importance of *Aeromonas*, mainly because this bacterium can cause diseases in animals and humans and because it presents high levels of antimicrobial resistance. Improvements in the management and breeding of ornamental fish must be implemented to reduce the need for drug treatment, and, in necessary cases, the conscious use of antimicrobials approved by veterinarians is essential to avoid further resistance dissemination and compromise of the antimicrobials used in human treatments.

## 5. Conclusions

Although the MALDI-TOF MS technique presented good results for the bacterial identification of several animal species, it still raises doubts regarding the appropriate species assignment within the *Aeromonas* genus that require molecular confirmation. Among the *Aeromonas* species detected, the *A. hydrophila* strains presented greater positivity for virulence genes, suggesting greater virulence potential. However, no relationship was observed between the virulence profiles and the clinical conditions or origins of the animals. Although genotyping by SE-AFLP showed greater heterogeneity for the *A. veronii* and *A. caviae* species, no direct relationship was observed between the genotypes and the origin of the strains or the virulence profile. Regarding antimicrobial resistance, sulfonamide and sulfamethoxazole-trimethoprim showed high resistance rates despite not being antimicrobials authorized for usage in fish. The high rate of multidrug resistance detected, especially for the species *A. caviae* and *A. hydrophila*, demands attention due to the risk of resistance dissemination in the aquatic environment and to the potential for compromising treatment in humans.

## Figures and Tables

**Table 1:** Identification of *Aeromonas* species by MALDI-TOF MS and PCR techniques—N (%).

ID PCR	ID MALDI-TOF MS			Total
	*A. veronii*	*A. hydrophila*	*A. caviae*	
*A. veronii*	141 (89.2)	18 (14.4)	0	159 (53.0)
*A. hydrophila*	16 (10.2)	103 (82.4)	5 (29.4)	124 (41.3)
*A. caviae*	1 (0.6)	4 (3.2)	12 (70.6)	17 (5.7)
Total	158 (100)	125 (100)	17 (100)	300 (100)

ID PCR—PCR species identification. ID MALDI-TOF MS—MALDI-TOF MS species identification.

**Table 2:** Distribution of infection profiles of *Aeromonas* species among the studied animals (N = 123).

Infection Profiles	Species	N (%)
P1	AVE	43 (35.0)
P2	AHY	37 (30.1)
P3	ACA	3 (2.4)
P4	AVE/AHY	29 (23.6)
P5	AVE/ACA	6 (4.9)
P6	AHY/ACA	3 (2.4)
P7	AVE/AHY/ACA	2 (1.6)

AVE—*A. veronii*; AHY—*A. hydrophila*; ACA—*A. caviae*.

**Table 3:** Frequency distribution of *Aeromonas* species according to the studied fish species—N (%).

Fish Species	*A. veronii*	*A. hydrophila*	*A. caviae*	Total
*Carassius auratus* (Linnaeus, 1758)	41 (52.6)	35 (44.9)	2 (2.5)	78 (100)
*Cyprinus carpio* var. koi	38 (53.5)	33 (46.5)	0	71 (100)
*Trichogaster trichopterus* (Pallas, 1770)	18 (64.3)	9 (32.1)	1 (3.6)	28 (100)
*Trichogaster lalius* (Hamilton, 1822)	1 (6.25)	14 (87.5)	1 (6.25)	16 (100)
*Mikrogeophagus altispinosus* (Haseman, 1911)	5 (33.3)	10 (66.7)	0	15 (100)
*Danio rerio* (Hamilton, 1822)	6 (66.7)	1 (11.1)	2 (22.2)	9 (100)
*Symphysodon discus* (Heckel, 1840)	6 (66.7)	2 (22.2)	1 (11.1)	9 (100)
*Cichlasoma salvini* (Günther, 1862)	6 (75.0)	0	2 (25.0)	8 (100)
*Barbodes semifasciolatus* (Günther, 1868)	4 (66.7)	2 (33.3)	0	6 (100)
*Cichlasoma meeki* (Brind, 1918)	1 (16.7)	2 (33.3)	3 (50.0)	6 (100)
*Pterophyllum scalare* (Schultze, 1823)	4 (66.6)	1 (16.7)	1 (16.7)	6 (100)
*Poecilia reticulata* (Peters, 1859)	2 (40.0)	3 (60.0)	0	5 (100)
*Trichopodus leerii* (Bleeker, 1852)	2 (40.0)	2 (40.0)	1 (20.0)	5 (100)
*Polypterus senegalus* (Cuvier, 1829)	4 (100)	0	0	4 (100)
*Puntigrus tetrazona* (Bleeker, 1855)	4 (100)	0	0	4 (100)
*Puntius conchonius* (Hamilton, 1822)	4 (100)	0	0	4 (100)
*Betta splendens* (Regan, 1910)	2 (66.7)	1 (33.3)	0	3 (100)
*Misgurnus anguillicaudatus* (Cantor, 1842)	2 (66.7)	0	1 (33.3)	3 (100)
*Pangasius hypophthalmus* (Sauvage, 1878)	2 (66.7)	1 (33.3)	0	3 (100)
*Trichopodus trichopterus* (Pallas, 1790)	2 (66.7)	1 (33.3)	0	3 (100)
*Thorichthys meeki* (Brind, 1918)	0	2 (100)	0	2 (100)
*Tropheus moorii* (Boulenger, 1898)	0	1 (50.0)	1 (50.0)	2 (100)
*Cyphotilapia frontosa* (Boulenger, 1906)	1 (100)	0	0	1 (100)
*Chindongo demasoni* (Konings, 1994)	1 (100)	0	0	1 (100)
*Epalzeorhynchos bicolor* (Smith, 1931)	0	0	1 (100)	1 (100)
*Hemiodus gracilis* (Günther, 1864)	0	1 (100)	0	1 (100)
*Hyphessobrycon herbertaxelrodi* (Géry, 1961)	1 (100)	0	0	1 (100)
*Maylandia zebra* (Boulenger, 1899)	1 (100)	0	0	1 (100)
*Melanotaenia trifasciata* (Rendahl, 1922)	0	1 (100)	0	1 (100)
*Moenkhausia costae* (Steindachner, 1907)	1 (100)	0	0	1 (100)
*Parambassis ranga* (Hamilton, 1822)	0	1 (100)	0	1 (100)
*Puntius tetrazona* (Bleeker, 1855)	0	1 (100)	0	1 (100)

**Table 4:** Frequency distribution of *Aeromonas* species according to the fish clinical condition—N (%).

Clinical Condition	*A. caviae* (N = 17)	*A. hydrophila* (N = 125)	*A. veronii* (N = 158)	Total (N = 300)
Septicemia	55 (34.6)	31 (25.0)	5 (29.4)	91 (30.3)
Ocular lesion and septicemia	48 (30.2)	35 (28.2)	7 (41.2)	90 (30.0)
Ocular lesion	15 (9.4)	30 (24.2)	3 (17.6)	48 (16.0)
Skin lesion	22 (13.8)	17 (13.7)	1 (5.9)	40 (13.3)
Skin lesion and septicemia	19 (11.9)	11 (8.9)	1 (5.9)	31 (10.3)

**Table 5:** Frequency distribution of identified virulence profiles according to *Aeromonas* species—N (%).

Profile	Virulence Profile	*A. caviae* (N = 17)	*A. hydrophila* (N = 125)	*A. veronii* (N = 158)	Total (N = 300)
V1	*hlyA−/aer+/act+/alt−/ast−/fla+*	0	11 (8.9)	71 (44.7)	82 (27.3)
V2	*hlyA+/aer+/act+/alt+/ast+/fla−*	0	49 (39.5)	1 (0.6)	50 (16.7)
V3	*hlyA−/aer+/act+/alt−/ast−/fla−*	1 (5.9)	5 (4.0)	40 (25.2)	46 (15.3)
V4	*hlyA+/aer+/act+/alt+/ast+/fla+*	0	30 (24.2)	1 (0.6)	31 (10.3)
V5	*hlyA+/aer−/act+/alt+/ast+/fla+*	0	6 (4.8)	12 (7.5)	18 (6.0)
V6	*hlyA+/aer−/act−/alt−/ast−/fla+*	6 (35.3)	2 (1.6)	0	8 (2.7)
V7	*hlyA−/aer−/act+/alt−/ast−/fla−*	0	1 (0.8)	6 (3.8)	7 (2.3)
V8	*hlyA−/aer+/act+/alt+/ast−/fla+*	0	0	6 (3.8)	6 (2.0)
V9	*hlyA+/aer+/act+/alt−/ast−/fla+*	2 (11.8)	3 (2.4)	1 (0.6)	6 (2.0)
V10	*hlyA−/aer−/act−/alt−/ast−/fla−*	1 (5.9)	0	4 (2.5)	5 (1.7)
V11	*hlyA−/aer−/act−/alt−/ast−/fla+*	3 (17.6)	1 (0.8)	1 (0.6)	5 (1.7)
V12	*hlyA−/aer−/act+/alt−/ast−/fla+*	1 (5.9)	1 (0.8)	3 (1.9)	5 (1.7)
V13	*hlyA+/aer−/act+/alt+/ast−/fla+*	0	3 (2.4)	1 (0.6)	4 (1.3)
V14	*hlyA+/aer+/act+/alt+/ast−/fla+*	0	2 (1.6)	2 (1.3)	4 (1.3)
V15	*hlyA−/aer+/act−/alt−/ast−/fla−*	0	1 (0.8)	2 (1.3)	3 (1.0)
V16	*hlyA−/aer+/act+/alt+/ast−/fla−*	0	0	3 (1.9)	3 (1.0)
V17	*hlyA−/aer−/act+/alt+/ast−/fla−*	0	0	2 (1.3)	2 (0.7)
V18	*hlyA+/aer−/act−/alt−/ast−/fla−*	2 (11.8)	0	0	2 (0.7)
V19	*hlyA+/aer−/act+/alt+/ast+/fla−*	0	1 (0.8)	1 (0.6)	2 (0.7)
V20	*hlyA−/aer−/act−/alt+/ast−/fla−*	0	1 (0.8)	0	1 (0.3)
V21	*hlyA−/aer−/act+/alt+/ast−/fla+*	0	1 (0.8)	0	1 (0.3)
V22	*hlyA−/aer−/act+/alt+/ast+/fla−*	0	1 (0.8)	0	1 (0.3)
V23	*hlyA−/aer+/act+/alt+/ast+/fla−*	0	1 (0.8)	0	1 (0.3)
V24	*hlyA−/aer+/act+/alt+/ast+/fla+*	0	0	1 (0.6)	1 (0.3)
V25	*hlyA+/aer−/act−/alt+/ast−/fla+*	0	0	1 (0.6)	1 (0.3)
V26	*hlyA+/aer+/act−/alt−/ast−/fla+*	1 (5.9)	0	0	1 (0.3)
V27	*hlyA+/aer+/act−/alt+/ast−/fla+*	0	1 (0.8)	0	1 (0.3)
V28	*hlyA+/aer+/act−/alt+/ast+/fla−*	0	1 (0.8)	0	1 (0.3)
V29	*hlyA+/aer+/act−/alt+/ast+/fla+*	0	1 (0.8)	0	1 (0.3)
V30	*hlyA+/aer+/act+/alt+/ast−/fla−*	0	1 (0.8)	0	1 (0.3)

**Table 6:** Frequency distribution of identified resistance profiles—N (%).

Antimicrobial	[µg] *	Susceptible	Intermediate	Resistant	Total
Cefoxitin	30	228 (97.4)	3 (1.3)	3 (1.3)	234 (100)
Ceftazidime	30	198 (84.6)	28 (12.0)	8 (3.4)	234 (100)
Ceftriaxone	30	214 (91.5)	16 (6.8)	4 (1.7)	234 (100)
Cefepime	30	179 (76.5)	50 (21.4)	5 (2.1)	234 (100)
Imipenem	10	102 (43.6)	98 (41.9)	34 (14.5)	234 (100)
Piperacillin-tazobactam	110	207 (88.5)	18 (7.7)	9 (3.8)	234 (100)
Chloramphenicol	30	208 (88.9)	22 (9.4)	4 (1.7)	234 (100)
Florfenicol	30	107 (45.7)	49 (20.9)	78 (33.4)	234 (100)
Tetracycline	30	172 (73.5)	36 (15.4)	26 (11.1)	234 (100)
Ciprofloxacin	5	110 (47.0)	89 (38.0)	35 (15.0)	234 (100)
Enrofloxacin	5	34 (14.5)	119 (50.9)	81 (34.6)	234 (100)
Sulfonamide	300	8 (3.4)	9 (3.9)	217 (92.7)	234 (100)
Trimethoprim-sulfamethoxazole	25	98 (41.9)	18 (7.7)	118 (50.4)	234 (100)
Erythromycin	15	5 (2.1)	175 (74.8)	54 (23.1)	234 (100)
Gentamicin	10	203 (86.8)	21 (9.0)	10 (4.2)	234 (100)
Amikacin	30	186 (79.5)	33 (14.1)	15 (6.4)	234 (100)

* [µg.]—Antimicrobials disc concentration.

**Table 7:** Frequency distribution of identified resistance profiles for florfenicol, gentamicin and enrofloxacin considering different cut-offs—N (%), (N = 234).

Classification		Florfenicol	Gentamicin	Enrofloxacin
ECOFF ^1^	Wild Type	75 (32.1)	108 (46.2)	0
	Non-Wild Type	159 (67.9)	126 (53.8)	234 (100)
CLSI VET 01S ^2^	Susceptible	107 (45.7)	203 (86.7)	34 (14.5)
	Intermediate	49 (20.9)	21 (9.0)	119 (50.9)
	Resistant	78 (33.4)	10 (4.3)	81 (34.6)

^1^ CLSI VET04—3rd ed [19]. ^2^ CLSI VET 01S—5th ed [23].

## Data Availability

The data presented in this study are available at the Zenodo repository using the following link: https://doi.org/10.5281/zenodo.10482713.

## Supplementary Materials

The following supporting information can be downloaded at: https://www.mdpi.com/article/10.3390/microorganisms12010176/s1. Table S1: Primers used to *Aeromonas* species identification and detection of virulence genes; Table S2: Antimicrobials evaluated and respective concentrations and applied cut-off points; Figure S1: Fish with clinical signs of ulcerative skin lesions (A—*Carassius auratus*, C—*Cyprinus carpio* var. koi), septicemia (B—*Cyprinus carpio* var. koi) and ocular lesion (D—*Carassius auratus* with exophthalmia and corneal opacity). References [13,14,19,20,23,48,49,50,51] are cited in supplementary file.
