# Supplementary material for: Characterization of Aeromonas Isolates from Ornamental Fish: Species, Virulence Genes, and Antimicrobial Susceptibility
Source: Microorganisms. 2024 Jan 16;12(1):176. doi: 10.3390/microorganisms12010176 (PMC10819562; doi:10.3390/microorganisms12010176)
Supplement: Supplementary file 1 [file microorganisms-12-00176-s001.zip › microorganisms-2813986-supplementary.pdf]

Supplementary material

Figure S1: Fish with clinical signs of ulcerative skin lesions (A - *Carassius auratus*, C - *Cyprinus carpio* var. koi), septicemia (B - *Cyprinus carpio* var. koi) and ocular lesion (D - *Carassius auratus* with exophthalmia and corneal opacity).

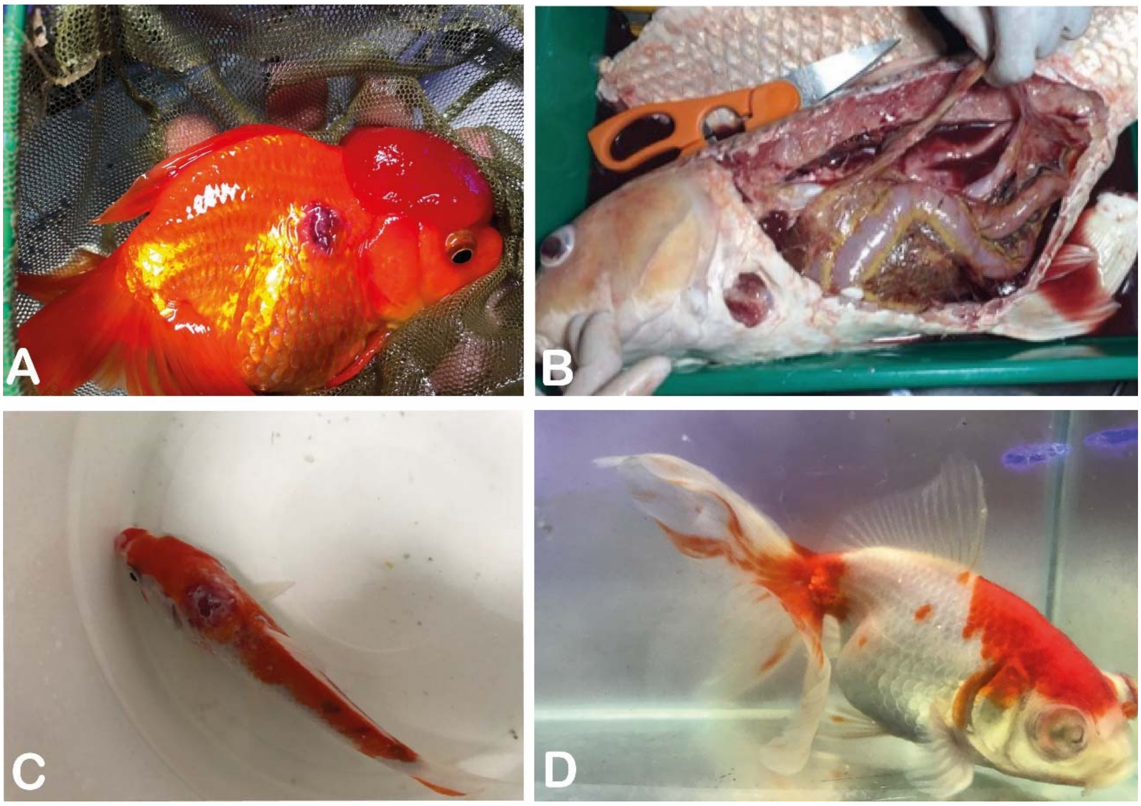

Table S1: Primers used to *Aeromonas* species identification and detection of virulence genes.

Gene	Target	Primer Sequence (5′ – 3′)	Amplicon (pb)	Reference
gyrB	*A. caviae*	TGCTGCTGACCATCCGC<br>GGTGCCTGCGGCTCG	70	[13]
ahaI	*A. hydrophila*	GAGAAGGTGACCACCAAGAACA<br>GAGATGTCAGCCTTGTAGAGCT	200	[14]
rpoB	*A. veronii*	CGTGCCGGCTTTGAAGTC<br>GATCACGTACTTGCCTTCTTCAATA	224	[13]
act	Cytotoxic enterotoxin	AGAAGGTGACCACCAAGAACA<br>AACTGACATCGGCCTTGAACTC	232	[48]
aer	Aerolysin	CCTATGGCCTGAGCGAGAAG<br>CCAGTTCCAGTCCCACCACT	431	[49]
alt	Heat-labile cytotonic enterotoxin	AAAGCGTCTGACAGCGAAGT<br>AGCGCATAGGCGTTCTCTT	320	[50]
ast	Heat-stable cytotonic enterotoxin	ATCGTCAGCGACAGCTTCTT<br>CTCATCCCTTGGCTTGTTGT	504	[50]
fla	Flagellin	TCCAACCGTYTGACCTC<br>GMYTGGTTGCGRATGGT	608	[49]
hlyA	Hemolysin	ATGAGTTTTGCCGATAGTTTATTTTTCCTGA<br>TTACGATTCCTGAGCGGGCTTGTCGGCCGGCG TG	1320	[51]

Table S2: Antimicrobials evaluated and respective concentrations and applied cutoff points.

Antimicrobial	Concentration (µg/mL)	CLSI breakpoints[1]		
		Susceptible	Intermediate	Resistant
Cefepime	30	≥25	19-24	≤18
Cefoxitin	30	≥18	15-17	≤14
Ceftazidime	30	≥21	18-20	≤17
Ceftriaxone	30	≥23	20-22	≤19
Imipenem	10	≥23	20-22	≤19
Piperacillin-tazobactam	110	≥21	18-20	≤17
Tetracycline	30	≥15	12-14	≤11
Enrofloxacin[2]	5	≥23	17-22	≤16
Ciprofloxacin	5	≥21	16-20	≤15
Amikacin	30	≥17	15-16	≤14
Gentamicin[2]	10	≥16	13-15	≤12
Erythromycin	15	≥23	14-22	≤13
Chloramphenicol	30	≥18	13-17	≤12
Florfenicol[2]	30	≥22	19-21	≤18
Sulfonamide	300	≥17	13-16	≤12
Trimethoprim-sulfamethoxazole	25	≥16	11-15	≤10

Antimicrobial	Concentration (µg/mL)	ECOFF cutoff points[3]	
		Wild Type	Non-Wild Type
Enrofloxacin	5	≥32	≤31
Florfenicol	30	≥25	≤24
Gentamicin	10	≥19	≤18

[1] CLSI - M100, 32nd ed [20]. [2] CLSI - VET 01S – 5th ed [23]. [3] CLSI - VET04, 3rd ed [19].
